# Supplementary material for: Are carbon emissions trading and green financial instruments synergistic? -Comprehensive quantitative research based on content analysis
Source: PLoS One. 2024 Mar 7;19(3):e0298601. doi: 10.1371/journal.pone.0298601 (PMC10919601; doi:10.1371/journal.pone.0298601)
Supplement: S1 Data — (ZIP) [file pone.0298601.s001.zip › Data/地级市Z_F工作报告.zip!/░▓╗╒/╬▀║■╩╨/└┤╫╘╣1⁄2╓┌║┼í╛┬φ┐╦╩2╛▌═°í┐_2017.docx]

政 府 工 作 报 告

——2017年1月9日在芜湖市第十五届人民代表大会第七次会议上

市长 潘朝晖

各位代表：

现在，我代表市人民政府，向大会报告政府工作，请予审议，并请市政协委员和其他列席人员提出意见。

一、2016年工作回顾

过去的一年，全市人民在中共芜湖市委的坚强领导下，认真学习贯彻习近平总书记系列重要讲话特别是视察安徽重要讲话精神，全面落实中央和省委、省政府的决策部署，主动适应经济发展新常态，着力推进供给侧结构性改革，促进民生改善，较好完成了市十五届人大六次会议确定的主要目标任务，实现了“十三五”良好开局。

全市生产总值2699.4亿元（预计数，下同），比上年增长9.7%；财政收入512.3亿元，增长9%；固定资产投资3006.9亿元，增长11%；社会消费品零售总额828.2亿元，增长13%；城乡居民人均可支配收入26160元，增长9%；城镇新增就业7.2万人、登记失业率3.3%；单位生产总值能耗下降4%；可吸入颗粒物年平均浓度下降6微克/立方米。

（一）经济保持平稳较快增长。出台了促进经济持续健康较快发展的34条意见。落实结构性减税等税收优惠政策，减轻企业负担86.4亿元，其中出口退税30.9亿元。发放稳岗和促进就业补贴2亿元，减缴缓缴社保费9.7亿元。通过直接交易降低工业用电成本2.2亿元。通过调低住房公积金缴存比例，降低企业成本1.1亿元。

加大金融支持实体经济力度。中国物流资产在港交所上市，众源新材、英派瑞2家企业报会，新增24家企业“新三板”挂牌。皖江金融租赁公司成为全省首家发债的非银金融机构。实现直接融资445亿元。金融机构净增贷款361亿元。通过政银担、续贷过桥、“税融通”等措施支持中小企业融资135.5亿元。应收账款质押融资突破200亿元。开展“订单贷”，缓解电线电缆行业融资难。累计设立基金150余只，总规模384亿元，初步形成覆盖企业种子期、初创期、成长期、成熟期的基金集群。

抓好招商引资和重大项目建设。实际利用省外资金1256亿元、外资25.3亿美元。在建亿元以上项目1151个，其中新开工511个。省“大新专”项目新开工246个，位居全省第一。民间投资增长11.9%，入选全省百强民营企业数位列全省第一。规模以上工业企业新增222户。中部地区首家中德（芜湖）中小企业合作区获批建设。中车单轨车辆及捷运系统胶轮车辆等项目开工。

（二）调结构转方式促升级取得新成效。出台供给侧结构性改革实施意见。改造提升传统产业，汽车及零部件、材料、电子电器、电线电缆四个支柱产业增加值增长11.8%。完成工业技术改造投资757.7亿元。航瑞动力等3家企业技改列入国家专项，鼎恒再制造等2家企业列入国家再制造试点。奇瑞新车型销售快速增长。海螺集团保持较高盈利水平。鲁班集团、宝翔集团晋升建筑工程施工总承包特级资质。

把握打造核心产业链、培育龙头企业、构建产业支持平台三个关键环节，战略性新兴产业增加值增长19.3%。通用航空产业基地被列为第二批省战略性新兴产业集聚发展试验基地，太赫兹芯片、肺动脉高压创新药物产业化及量子通信被列入第一批省新兴产业重大工程、重大专项。在国内率先形成机器人全产业链集聚发展态势。埃夫特公司收购意大利EVOLUT公司，六关节机器人销量居全国首位。瑞思等机器人项目实现量产。加拿大扎巴科机器人、上海酷哇机器人、机器人孵化器二期等项目开工。国家工业机器人产品质量监督检验中心（安徽）获批筹建。新型显示及光电信息产业链更加健全。重点发展玻璃基板等上游配套关键原材料和生产设备领域，完善有机材料等配套产业环节，实现产业链关键环节的技术和产业化突破。太赫兹工程中心、赋兴光电摄像头模组等项目开工。新能源汽车产业快速推进。奇瑞新能源公司全铝车身项目一期已建成，二期已开工。奇达动力电池系统已投产，天弋锂电池二期逐步投产。奇瑞、宝骐新能源汽车销售比上年翻一番。奇瑞百度首款无人车亮相“十二五”科技成果展。现代农机产业稳步推进。中联重机公司烘干机、小麦机、水稻机销量居全国前列，甘蔗机突破机械化收获瓶颈。羽人农业航空、三普智能重工挖树机、中农智全地形植保车等项目投产，新建特种农业装备、中机精密成形2个产业技术研究院。通用航空产业加速集聚。中电科钻石飞机项目一期建成，首架“安徽造”飞机下线。航瑞动力公司研发6款航空发动机及1款纯电动涵道风扇推进系统，填补国内行业空白。钻石航空发动机项目基本建成。新材料产业加快布局。格丰纳米新材料、格力精密铸造等项目投产，安美利特新材料、长青藤新材料等项目开工。

服务业实现增加值1060.8亿元，增长11%。出台加快发展生活性服务业、促进消费结构升级、贯彻省“十三五”物流业发展规划的实施意见。华夏银行芜湖分行开业。中国平安综合金融电商物流园开工，共生物流平台交易额超10亿元。网上商品零售额比上年翻一番，三只松鼠、凡臣电子公司销售收入增长迅猛。快递业务量增长82%。方特旅游区获批国家5A级景区。奇瑞工业园获评首批国家工业旅游创新单位。南陵县成为全国休闲农业和乡村旅游示范县，丫山获批国家地质公园、国家牡丹公园。陶辛水韵获批国家水利风景区和4A级景区。新华联鸠兹古镇、大白鲸海洋公园建成开业。接待游客3900万人次，增长18%。服务外包执行金额14.2亿美元，增长15%。成功举办第七届中国（芜湖）科博会、中国机器人产业推进大会、第五届刘开渠奖国际雕塑大展等重大会展。

推进农业现代化。农作物耕种收综合机械化水平达66%。新增家庭农场951个、农民专业合作社201个。溜溜果园、益海嘉里、古麒羽绒、东隆羽绒等龙头企业发展加快，农产品加工产值超600亿元。阡陌科技率先打造覆盖全国的三农互联网服务业平台。新增认证无公害农产品61个、绿色食品60个、有机食品10个、地理标志1个。

（三）改革开放迈出新步伐。建成市县乡三级公共服务清单体系。完成三级政府权责清单“六统一”。 在全省率先公布市级政府部门行政审批中介服务清单，清理和取消不合理的中介服务事项。深化行政审批制度改革，及时承接国务院、省政府取消和调整行政审批项目等事项，持续优化重大项目服务，完善工作联动机制。清理规范行政权力和公共服务事项所涉证明材料。市级涉企收费项目精简25%。开展“互联网+政务服务”，市大数据中心共享汇集60个政府部门、220亿条数据信息。市级4773项、各县区20493项行政权力和公共服务事项全面实现网上运行，当年办理各类事项43万件，其中行政许可通过易户网、易企网等网上申请办理占20%，规定时间办理率和群众满意率达99%。社会信用体系示范城市建设通过国家中期评估。行政处罚信息公示数、统一社会信用代码存量转换率位列全国第一。在全省率先启动“五证合一”登记制度改革，探索开展企业简易注销登记改革。全省首张电子营业执照在芜发放。开展以市场监管领域为重点的综合监管、分类执法改革试点，获国务院通报表扬。加快医药卫生体制改革，加强对公立医院运营监管，开展家庭医生签约服务，推行分级诊疗制度。整合建立市县一体化公共资源交易平台。开展物流标准化试点，编制中国（芜湖）托盘指数。农村土地承包经营权确权登记颁证试点工作基本完成。农村集体资产股份制改革试点积极推进，国有林场改革按期完成。市县不动产统一登记全面实施。推进工业用地市场化配置改革试点、矿产资源节约集约开发利用试点和农村产权交易体系建设试点。我市成为全国国土资源节约集约模范市。

清理盘活财政存量资金10.9亿元。争取政府置换债券107.3亿元、新增债券26.4亿元。完善国地税征管体制，“营改增”税制实现平稳转换。完善市区经营性国有土地使用权收支管理体制，启动中期财政规划管理。出台深化国资国企改革的实施意见。

出台贯彻长三角城市群发展规划、构建开放型经济新体制的实施意见。江北产业集中区总体规划获省政府批复。新芜经济开发区获批长江经济带国家级转型升级示范开发区。综合保税区跨境电子商务产业园一期竣工，跨境电子商务平台建成运营。复制推广自贸试验区改革事项35项。港口货物吞吐量1.3亿吨，增长9.1%；集装箱吞吐量60.2万标箱，增长20%。实现进出口总值375.9亿元。推进芜湖港与上海港及周边港口深度合作，芜宣“无水港”开港运营。中铁特货商品汽车中转物流基地落户芜湖港。新增实际对外投资5.7亿美元。

（四）城市规划建设水平进一步提升。完成了城市公共服务设施、公交、中心城区慢行系统规划编制和历史文化名城规划方案。完成了中心城区轨道交通沿线地区土地开发利用、港产城融合发展等课题研究。开展了永久基本农田、城市开发边界和生态红线的统筹划定，形成“多规合一”控制线体系。完成了弋江路景观及绿化提升等项目设计。启动城市规划实施动态监控试点。

着力改善交通。芜湖宣城民用机场试验段、城市轨道交通1号线及2号线一期、商合杭铁路长江大桥接线、火车站西站房、弋江路米市口立交和马塘立交、中山桥和中江桥改建、329国道芜湖县段改造、淮九路改建等工程开工。澛港闸大桥、205国道南陵段快速化、321省道改造、弋江路赤铸山路立交、和谐大道、无六路等工程建成通车，205国道市区段改建基本完成。完成农村道路畅通工程1078公里。建成149个新能源汽车租赁点、3004根充电桩位、3311个停车位，构建芜湖新出行模式。青弋江分洪道全线贯通，芜申运河航道工程基本完成。安徽中桩物流码头、东汇三山码头、皖江物流裕溪口码头及富航物流码头等投入使用。渡口渡船升级改造工作基本完成。

优化城市环境。棚户区改造完成2.7万户。商品房销售736.9万平方米，增长21.9%。实施市区存量房交易网签合同备案。启动城市出入口及交通干线品质提升工程。完成“三线三边”整治任务，实施“城镇大绿化”提升工程，新增绿地445万平方米，新建绿道95.6公里。神山公园亮化、汀棠公园景观、板城垾截污及景观整治工程一期竣工。中江桥至袁泽桥段防洪墙综合改造、青弋江治理工程开工。探索制定市级绿色矿山标准，治理矿山18家。芜湖发电公司20兆瓦光伏电站建成。市备用水源工程竣工。长江路地下综合管廊开工，推进污水设施统一管理和地下管线普查。完成重点减排项目39个。饮用水水源达标率100%。完成12条黑臭水体和39条市区明渠清淤清障整治，农村沟渠湖塘清淤疏浚成效明显。完成20个乡镇建成区整治和17个省级美丽乡村中心村建设。淘汰黄标车、老旧车1.3万辆。建成餐厨垃圾处理厂，开展餐厨垃圾集中收运与无害化处置。加强物业项目综合验收和备案。制定老旧小区综合整治实施方案，整治老旧小区43个。开展市民文明素质和城市文明水平“双提升”活动。国家森林城市创建启动，我市荣膺“中国雕塑之城”。繁昌县获批国家级循环经济示范县。

（五）创新创业活力持续增强。出台了系统推进全面创新改革试验实施方案、推进国家自主创新示范区建设工作方案、发展众创空间促进大众创业万众创新的实施意见。新增省级“专、精、特、新”企业50个。新认定3家国家级众创空间，2个省级青年创业园，3个省级小微企业创业创新示范基地。芜湖高新技术创业服务中心获批全国百强科技企业孵化器。各类市场主体达21.3万户，增长16.8%。

兑付科技创新系列政策资金2.5亿元。高新技术产业增加值815.8亿元，增长17.5%。新认定高新技术企业114家。万人有效发明专利拥有量达21件，稳居全省第一。高新区获批国家科技服务业区域试点。新增省级重点实验室、省级工程技术研究中心9家，省级以上企业技术中心15个，省级行业技术中心2个，院士工作站6家，博士后科研工作站3家，重点产业技术研究院3家。推进质量品牌升级工程，奇瑞荣获省政府质量奖，新增中国驰名商标4件。

（六）社会各项事业全面进步。33项民生工程完成投入99.6亿元。出台了坚决打赢脱贫攻坚战的实施意见，大力实施精准扶贫、精准脱贫，投入3亿元扶贫资金，实现3万贫困人口脱贫，34个贫困村出列。完善了基本养老、医疗、失业、工伤、生育保险及大病医疗补充保险制度，社会保障覆盖面进一步扩大。企业基本养老金由1816元提高到1895元。推动医疗、工伤保险支付方式改革，新型农村合作医疗保险政策范围内报销比达75%。完成就业技能培训1.8万人、岗位技能提升培训0.9万人，新增省级技能大师工作室1家。为12.5万低保对象发放低保金5.1亿元。发放3.3万人次困难群众医疗救助款8873万元。住房保障达8.5万户。住房公积金缴存人数新增6.8万人，支持1.6万户家庭购房贷款。进城务工农民缴存住房公积金试点推广实现全覆盖。

深入推进教育名城建设，新成立教育集团6个，新设安师大附小东西校区、北师大附属幼儿园等优质学校分校（分园）13个，华师大芜湖外国语学校建成招生。新成立名校长工作室20个。完成66所学校标准化建设。安徽工程大学机电学院转设为安徽信息工程学院。市文化馆和芜湖县、繁昌县、南陵县、镜湖区文化馆被评为国家一级文化馆；出台了扶持地方戏曲发展政策，启动地方戏曲普查；9个文化企业获批全省文化影视出版示范园区（基地）。推行基层慢性病用药福利管理，开展了国家精神卫生综合管理试点。编制了养老设施布局规划，我市成为第一批国家级医养结合试点单位。实施“全面两孩”政策，深化计划生育服务管理，推行卫生计生“七证合一”。实施社会组织信用代码，完成1669家社会组织新证换发。全面实施食品药品风险分级监督管理，成功创建全省首批食品药品安全城市。出台了加强城乡社区协商、推进农村社区建设工作的实施意见，推进“互联网+社区公共服务”。开展法律援助服务5.3万人次。支持工会、共青团、妇联等群团组织发挥作用。民族、宗教、外事、侨务、对台、人防、老龄、残疾人、档案等工作进一步发展。创新双拥制度，落实优抚安置政策，市烈士陵园入选国家级烈士纪念设施。巩固“铸安”行动成效，加大安全生产事故查处和问责力度。强化危化品、非煤矿山、燃气和交通、建筑等领域安全检查，完成14处地质灾害隐患点治理。深入推进“平安芜湖”建设，健全治安防控和处置机制，严厉打击违法犯罪，社会治安和信访形势稳定。

（七）依法行政能力不断增强。政府系统开展了“两学一做”学习教育和“讲看齐、见行动”学习讨论。推进重大事项合法性审查机制全覆盖和重大行政执法决定法制审核制度，落实行政执法资格管理制度。开展了规范性文件清理，实施招商引资合同履约监管，进一步规范重大招商及投资项目评估决策。深入推进“亲切服务”专项行动。办理人大代表建议158件和政协提案277件。强化审计监督，加强行政监察，保持惩治腐败高压态势不动摇。

各位代表，去年我市遭遇了仅次于1954年的特大洪水袭击，面对罕见的自然灾害，全市广大干部群众和部队官兵不惧艰险、不怕牺牲、严防死守、抢险救灾，夺取了抗洪抢险全面胜利，涌现出王能珍等一大批可歌可泣的先进典型。目前，灾后恢复重建任务基本完成。在这场斗争中，全市上下展示出万众一心、众志成城，不怕困难、顽强拼搏，坚韧不拔、敢于胜利的伟大抗洪精神。我们要倍加珍惜，大力弘扬，并将其转化为助推芜湖发展的强大动力。

各位代表，在经济下行压力较大、困难挑战增多、洪涝灾害突发的情况下，去年的成绩来之不易。这是党中央、国务院、省委、省政府和市委正确领导的结果，是全市人民团结奋斗、苦干实干的结果。在此，我代表市人民政府，向全市人民，向各民主党派、各人民团体和各界人士，向驻芜解放军指战员、武警官兵和政法公安干警，向关心、支持芜湖改革发展的上级部门、兄弟市、企事业单位和海内外友好人士，表示衷心的感谢！

同时，我们也清醒认识到，当前发展还存在不少问题，主要是：优化经济结构的任务仍然艰巨，战略性新兴产业还处在培育发展阶段，服务业层次和水平还需提高。城市规划、建设水平不够高，管理不够科学、精细。政府职能转变还不到位，少数政府部门和工作人员服务意识、责任意识、担当精神不强，对群众和基层反映的问题不能及时有效解决，推诿扯皮等现象时有发生。资源环境约束加剧，市民反应强烈的占道经营、交通乱象等问题仍然突出。民生工作和社会治理存在薄弱环节，教育、医疗发展仍存在不均衡问题，养老服务业发展较为缓慢，部分群众生活困难。对此，我们一定高度重视，切实采取有效措施，认真加以解决。

二、2017年工作总体部署

今年是实施“十三五”规划的重要一年，是供给侧结构性改革的深化之年，也是本届政府任期最后一年。我们要恪尽职守、决不懈怠，保持经济社会平稳健康发展，交出一份人民满意的答卷。

当前，世界经济进入发展转型期，国际分工、产业竞争不断深化，新一轮科技和产业革命如火如荼。我国经济发展新常态特征更加明显，经济发展方式加快转变，新的增长动能正在孕育形成，全面深化改革红利持续释放，大众创业、万众创新热情充分激发。国家实施三大发展战略，“全创改”、“合芜蚌”等多个国家级平台在芜湖交汇叠加。经过近年来特别是“十二五”发展，我市传统产业转型升级成效明显，战略性新兴产业和服务业加快发展格局基本形成，以高端人才为核心的创新资源加速集聚，一批重点产业和重大项目快速成长、逐步发力，经济发展内生动力日益增强。我们要坚定信心，乘势而上，当好皖江崛起排头兵、五大发展先行者。

今年政府工作的总体要求是：全面贯彻党的十八大和十八届三中、四中、五中、六中全会精神，深入贯彻习近平总书记系列重要讲话特别是视察安徽重要讲话精神，认真落实党中央和国务院、省委、省政府、市委决策部署，坚持稳中求进工作总基调，坚持以新发展理念统领发展全局，适应把握引领经济发展新常态，以推进供给侧结构性改革为主线，以实施五大发展行动计划为总抓手，坚持“向东看、往东比”，紧紧抓住系统推进全面创新改革试验、国家自主创新示范区建设机遇，深入推进人才优先发展主战略，打造经济、城市两个升级版，促进人才结构高端化、产业层次高端化、城市品质高端化良性互动，建设创新协调绿色开放共享新芜湖，以优异成绩迎接党的十九大胜利召开。

今年经济社会发展的主要预期目标是：全市生产总值增长9.5%以上，财政收入增长9%，居民可支配收入增长9%，城镇新增就业6.5万人、登记失业率控制在4.5%以内，节能减排完成省政府下达任务。

全市上下必须以开阔的视野、广阔的胸襟，树立更加高远的目标，作更加长远的谋划，力争更大的作为。要把握以下原则：一是更加注重科技引领、人才优先。深入推进创新型城市、人才特区、创业特区建设，加快构建现代产业体系，推动芜湖制造率先迈向中高端。以系统思维、科学思维和担当精神谋划推进各领域改革，释放发展活力和动力。二是更加注重城乡统筹、拥江联动。树立全域理念、联动思维和精品意识，坚持新型工业化、信息化、城镇化、农业现代化同步发展，聚焦提升市区和县城品质，科学统筹新型集镇和美丽乡村建设，全面提高城乡规划建设管理水平。深化长三角战略合作及省内城市联动发展。三是更加注重生态环保、宜居宜业。坚持绿水青山就是金山银山的理念，大力发展绿色低碳循环经济，加强环境保护和生态建设，构建绿色发展方式和生活方式。四是更加注重内外并举、双向互动。坚持对内开放与对外开放并重、“引进来”与“走出去”并重，推进开放发展，构建法治化、市场化、国际化的营商环境，打造内陆开放新高地。五是更加注重民生为本、惠民利民。在做大“蛋糕”的同时分好“蛋糕”，把精准脱贫摆在首要位置，集中力量加强基础性、兜底性民生建设，推进基本公共服务均等化，使改革发展成果更多更公平惠及全市人民，着力增强人民群众获得感。

三、2017年重点工作

（一）着力构建创新型现代产业体系。按照战略性新兴产业能快则快、传统产业加速转型升级、优势产业迅速做大做强的思路，完善产业体系。出台“十三五”战略性新兴产业发展规划，聚焦战略性新兴产业重大基地、重大工程、重大专项建设。扩大机器人及智能装备、新型显示及光电信息、新能源汽车、现代农机、通用航空产业规模实力，培育节能环保、新材料、生命健康、轨道交通装备等产业基地。扶持龙头企业做大做强，围绕龙头企业打造上下游全产业链，提升产业核心竞争力。加快太赫兹工程中心、长江医学科学院、国家工业机器人产品质量监督检验中心（安徽）、安川电机、忠旺铝合金精深加工、智慧农业示范区、中小型航空发动机、海创节能板材二期等项目建设。

推动传统产业改造提升。落实稳增长政策措施。支持奇瑞、海螺、美的、新兴铸管等龙头企业发展，加速发展汽车电子、光缆、智能家居、新型材料等产业，引领带动支柱产业迈向中高端。组织实施100个重点技术改造项目，鼓励采用新技术、新工艺、新设备改造提升传统产业。积极推广节能工艺技术装备，增强企业绿色精益制造能力。推动制造业与大数据、物联网、工业设计深度融合。分类处置“僵尸企业”，鼓励企业通过战略合作、资本运作等方式做大做强。依靠市场力量，将转型升级与淘汰落后产能相结合，启动50家企业兼并重组。支持南陵打造中国建筑之乡。

大力发展现代服务业。以推动服务业发展提速、比重提高、水平提升为重点，加快实施服务业重大增长点“6115”工程。重点发展金融、现代物流、电子商务、文化旅游、服务外包等现代服务业，推动生产性服务业向专业化和价值链高端延伸，生活性服务业向精细化和高品质提升。新增1-2家银行业金融机构，力争民营银行、消费金融公司获批。支持以方特、新华联为龙头的城东文化旅游产业聚集发展，加快芜湖古城、芜湖旅游集散中心等项目建设。办好第五届中国国际动漫创意产业交易会。争创国家文化产业示范园、国家文化消费试点城市。培育互联网、大数据、云计算产业，加快“老船厂·智慧港”产业园建设，支持共生物流、阡陌科技、三只松鼠、三七互娱等企业做大做强。积极申报省级现代服务业集聚区。

深入推进农业供给侧结构性改革。把增加绿色优质农产品供给放在突出位置，狠抓农产品标准化生产、品牌创建、质量安全监管。积极培育新型农业经营主体和服务主体。持续推进现代农业示范园区建设。推动畜牧业转型升级。鼓励发展高科技设施农业、绿色生态农业、休闲观光农业。推进休闲农业与乡村旅游示范县（乡镇）创建。建设安徽农业大数据中心，推动智慧农业应用，加快农业机械化、信息化。推进农机农艺农信融合发展。深化农村集体资产股份制和“三资”改革试点。

（二）全力促进经济提质提速增效。坚持新产业、新技术、新业态、新模式加资本市场的发展思路，深入推进“三去一降一补”。强化“一企一组一策”帮扶机制，推动企业降本增效。以提高质量和核心竞争力为中心，深入推进质量品牌升级工程，着力振兴实体经济。继续化解过剩产能。着力降低企业杠杆率，持续推进企业对接多层次资本市场，落实政银担、“税融通”、续贷过桥等金融支持实体经济发展政策。深化金融与科技融合发展，建设互联网金融在线综合监管平台、中小企业信用贷平台，谋划建设长江航运交易所。力争1-2家企业首发上市、5家企业报会，新增“新三板”挂牌企业20家，完成直接融资300亿元。在减税、降费、降低要素成本上加大工作力度，支持企业降成本。优化政务服务，营造民营经济发展良好氛围。

牢固树立投资环境是城市最核心竞争力的意识，一丝不苟、精益求精地做好企业投资发展服务工作，全力塑造芜湖最佳投资地的品牌形象。提升产业培育科学化水平，由单纯依赖招商引资向系统培育和振兴产业转变，做到招商引资与招才引智并重、招引新企业与鼓励存量企业技改扩张并重、引进企业与孵化企业并重，扩大有效投资。县区（开发区）要聚焦重点产业，精准发力、高位起跳。深化“招大商、引巨资”意识，发挥国家级经济技术开发区、国家高新技术产业开发区、综合保税区和省级开发区平台作用，谋划、招引、培育一批重大项目。严格落实“四督四保”制度，确保项目建设扎实推进。

（三）深入实施人才优先发展主战略。按照人才特区建设总体部署，面向全球广泛招引高端创新创业团队和人才。完善人才政策，多措并举留住、用好人才。力争引进高层次科技人才团队60个以上，积极争取国家、省人才计划支持。

推进国家自主创新示范区建设。鼓励企业加大研发投入，加快研发机构建设，提升创新能力。支持新型研发机构建设，引导高校、研发机构和社会资本参加，打造共性技术、关键技术的公共研发平台，增强产业竞争力。提升知识产权创造质量。完善政产学研合作机制，建立成果转化激励机制，释放科技人员创新创业活力，促进科技成果转化和产业化。深化科技管理体制改革。

推进有芜湖特色的“双创”，营造全国一流的“双创”环境。发挥科技企业孵化器、创业投资机构、高校、科研机构的作用，支持创新工场、创客空间等各类众创空间发展。扩大政府投资引导基金规模，引进培育天使投资基金，缓解创业人才的融资难题。丰富活动形式，构建灵活多样的创客交流平台。开展常态化的创业集训营、创新创业赛事等活动，不断活跃城市创新创业氛围。

（四）全面提升城市建设和管理水平。坚持在宜居宜业品质上与一线城市等高对接，加快“山水相间、精致繁华”的城市风貌特色塑造。优化城市功能布局。推进城市规划实施动态监控试点，完善配套管理制度。推动各县加快编制“多规合一”规划。结合城市出入口及重要交通干道品质提升要求，推动城市设计向城市重点区延伸。加强控制性详细规划编制，继续推动单元规划及地块图则编制。

完善城市交通体系。推进民用机场、城市轨道交通建设。完成长江二桥、庐铜铁路芜湖段、火车站西站房、北沿江高速巢无段、弋江路米市口立交、沿江高速峨山路互通、芜合高速二坝（长安）互通立交改造、通江大道北延线（含裕溪河大桥）、黄山东路东延、205国道南陵渡大桥改建、弋牧公路等工程。开工建设芜合高速二坝至林头段改扩建、芜黄高速、国道205沿江高速芜湖南出入口互通立交和芜湖东高速出入口改建、八里湾大桥改建、峨山路东延二期、通江大道市区城建段、红花山路、徽州路城建等工程。全面启动市区快速路网建设。支持无为高铁站扩容改造。谋划推进合芜宣城铁项目。申报国家“公交都市”创建城市。规范公交车、出租车、网约车、公共自行车管理，推进公共停车场建设运营。加快中江桥、中山桥改建，完成芜申运河航道整治，配合整治合裕线航道。加快华谊化工码头还建、中石化芜湖油库码头迁建、江北集中区公用码头二期、中外运三山码头二期、中铁十四局芜湖大盾构基地码头等工程进度。

加大城市建设管理力度。实施弋江路、九华路、高铁沿线、长江路、万春路等品质提升工程。举办第六届刘开渠奖国际雕塑大展。继续推进高层住宅二次供水改造。推进江北新区供气设施建设。开展城市地下管网普查。建成长江路地下综合管廊。推进长江芜湖段综合整治、芜湖闸、神塘河大型排涝泵站等前期工作。加快青弋江治理、裕溪河治理、无为县五千亩以上圩口达标和袁泽桥、林都圩大中型排涝泵站等工程建设，完成双摆渡、荆方大型排涝泵站和青弋江城区段防洪综合治理、中小河流治理等项目，清疏下水道，整治明渠，增强水利工程抗灾能力，推进地质灾害隐患点治理，确保城乡安全度汛。加快华电二期100万千瓦超超临界机组项目和电网、天然气等基础设施项目建设，推进LNG（液化天然气）船舶改造和江海联运试点，完成农网改造项目。改造棚户区2.3万户。把房地产去库存与促进人口城镇化结合起来，加强市场监测，促进房地产市场平稳健康发展。调整完善棚改安置方式，推进棚改工作提速提效。建立健全公租房与安置房并轨使用制度。加强房地产开发项目和建筑工程安全质量监管。确保违法建设零增长。扩大全国文明城市创建成果。深入开展市容环境综合整治，加快环卫设施建设，实行农村卫生保洁和垃圾收运市场化。持续推动美丽乡村建设。完成城区在建工地建筑垃圾运输车的升级换代。启用建筑垃圾智慧管理系统，完成全市建筑垃圾消纳场规划选址。推进老旧小区、老旧市场改造升级，提升城市美化、亮化水平。加强对物业管理企业的监督问效。

牢固树立全市“一盘棋”的思想，高度重视江北发展，在基础设施、产业培育、民生保障等方面予以倾斜，引导江北聚焦发展、聚力突破。支持无为、南陵建设中等规模城市。加快芜湖县、繁昌县融入市区。推进新型城镇化试点、农村产权交易体系建设试点，提升城镇落户便利性。推广无为县城乡供水、供气、污水处理、垃圾处理、公共交通“五统一”运营模式。

（五）不断深化改革开放。贯彻《芜湖市系统推进全面创新改革试验实施方案》，努力建设成为长江经济带产业创新中心，力争以专利技术、人才保有量为核心的发展质量指标进入长三角第一方阵，经济总量指标进入全国地级市第一方阵。深化商事制度改革，积极探索“多证合一”，推进注册登记全程电子化。深化综合监管、分类执法改革，大力推行“双随机、一公开”，构建具有芜湖特色的事中事后监管新模式。加快大数据建设，部门各负其责，做到各数据库全面、准确、及时更新，推进部门协同和技术、数据、业务融合。深化“互联网+政务服务”，推进全市行政权力和公共服务事项“六统一”网上运行，打造“政务服务一张网”。协同完善网格化城市管理、社会管理和市场监管平台服务功能，构建闭环运行的城市治理体系。完成城市管理执法体制改革。深化公共资源交易改革。深化国资国企改革，促进民营经济发展。实行最严格的政府负债管理，规范政府举债行为，降低融资成本。实现城市卡全覆盖。深化公立医院改革，构建医疗质控体系和公立医院运营监管体系，持续推进分级诊疗、家庭医生签约服务。开展全域永久基本农田划定工作。完成不动产信息平台二期建设。

推进更深层次更高水平的双向开放。复制推广自贸试验区经验，积极申建自贸试验区。大力培育出口导向型企业，加快形成出口基地集群。积极申报进口指定口岸。加快发展跨境电子商务，推进进口商品直销中心和期货保税交割平台建设。促进合芜发展带、芜马宣城市组群建设，依托民用机场谋划空港经济区。加强芜湖港与相关港口的合作，持续推进航运服务业集聚区建设，强化芜湖物流枢纽地位。加快中德（芜湖）中小企业合作区发展，鼓励企业参与“一带一路”建设，深化对内对外开放合作。

（六）大力推进生态文明建设。树立“像保护眼睛一样保护生态环境，像对待生命一样对待生态环境”的理念，坚决向污染宣战，把环境保护摆在各项工作的突出位置。落实排污许可证、区域环境空气质量考核机制，加大治水、治气、治土工作力度，减少主要污染物排放总量。清理水源地违法建设，尽早实施水体达标方案。巩固钢铁水泥行业、扬尘、燃煤小锅炉、餐饮油烟、秸秆焚烧等重点大气污染源整治成果。稳步开展土壤污染普查和风险评估。强化工业集中区污染治理。提高绿洲环保能源公司、城南生活垃圾焚烧厂、餐厨垃圾处理厂的垃圾处理能力，实现日产生活垃圾全部焚烧、餐厨垃圾集中收运与无害化处置。加快雨污分流，完善污水管网体系，实施朱家桥、城南、滨江、天门山污水处理厂提标改造。

严格落实水资源管理制度、国土资源管理共同责任机制，加强水、土地、矿产等资源节约集约利用。落实主体功能区规划，加强生态空间管控。节约集约利用每一寸土地，清理闲置和低效利用土地，加大土地开发复垦整理、城乡建设用地增减挂钩、工矿废弃地复垦利用推进力度。大幅提升土地复垦指标补贴额度。加强地质环境保护治理，推进绿色矿山建设。严禁新建高耗能高污染和产能过剩项目。推进园区循环化改造，发展低碳循环经济。

改善生态环境。以创建国家森林城市、生态园林城市和水生态文明城市为抓手，实施“增绿增效”行动，全面扩大绿量，提高植绿质量，让全市域绿起来、美起来。建设滨江公园二期、汀棠公园二期和中央公园二期，提升桥头公园品质，加强公园管理。新增绿地350万平方米，新建绿道60公里。完成造林5.2万亩。实施保兴垾环境综合整治。加快城市黑臭水体整治。建设扁担河至大阳垾补水泵站。加快龙窝湖湿地公园前期工作。

（七）持续推动民生改善和社会事业发展。深入践行以人民为中心的发展思想，多谋民生之利，多解民生之忧。投入108.5亿元，实施30项以上民生工程，补好民生短板。加强民生工程管理。

扎实推进就业创业。成立市众创空间联盟，完善创业担保贷款运行机制。发挥芜湖高校毕业生就业联盟品牌效应，加大高校毕业生“免费租房、购房补贴、就业直补”等一揽子政策的落实力度。出台技工强市的实施意见。加快皖江人力资源公共实训基地建设。开发公益性岗位，安置就业困难人员。

实施全民参保登记计划，推进社会保险法定人群全覆盖。全面实施机关事业单位养老保险制度改革，建立健全基本社会养老保险体系。建立统一的城乡居民基本医疗保险制度，实现覆盖范围、筹资政策、保障待遇、医保目录、定点管理、基金管理“六统一”。发展慈善事业，加强社会救助。

合理配置和优化提升教育医疗资源，促进集团化、规范化、优质化发展。实现所有义务教育学校基本达标，加快推进城市新区中小学校建设。实施第三期学前教育行动计划。推动高中教育优质、特色、多样化发展。强化职业教育资源整合和市级统筹，实施中职教育质量提升工程，深化校企合作，推进中德职业教育合作。发展老年教育。全面落实乡村教师支持计划，努力建立结构合理、稳定的中小学教师队伍。支持在芜高校发展，支持国际工程师学院建设。实施“全面两孩”政策，推进计划生育转型发展。加强医保管理，打击骗保行为，遏制小病大治、过度医疗等现象。推进弋矶山医院三山医养结合示范园区、市妇幼保健计划生育服务中心建设，建成皖南精神卫生中心。积极创建省级卫生城市。

开展精准扶贫、精准脱贫。落实定点帮扶机制，完善脱贫攻坚政策体系，实施产业脱贫、就业脱贫、智力扶贫、社保兜底脱贫、健康脱贫、基础设施建设扶贫、金融扶贫、社会扶贫八大脱贫攻坚工程，动员各方力量，坚决打赢脱贫攻坚战。

加大公共事业投入。启动市文化活动中心项目建设，推进市文化馆改扩建项目和图书阅读点建设。打造“欢乐芜湖·百姓大舞台”系列文化品牌。贯彻芜湖铁画保护和发展条例，筹建铁画博物馆。加快文学艺术、新闻出版、广播电视、档案、地方志等事业发展。启动创建全国数字档案馆。完善体育设施，建设市游泳馆，发展全民健身运动，举办市第十三届运动会。做好第三次全国农业普查登记。发展人防、气象、防灾减灾等各项事业。提升养老服务设施水平，建设多层次养老服务体系。支持残疾人事业发展。做好民族、宗教、外事、侨务、科普等工作，推进少数民族流动人口服务管理示范城市建设。支持群团组织、社会组织履行职责。深入开展“七五”普法。着力增强全民国防意识，加强国防教育、优抚安置和双拥共建，支持驻芜部队建设。

牢固树立发展决不能以牺牲安全为代价的红线意识，贯彻安全第一、预防为主、综合治理的方针。落实党政同责、一岗双责、齐抓共管、失职追责，完善安全生产责任体系。加强智慧社区建设，把公共安全防范的关口前移，建立健全安全风险防控和隐患治理工作机制。强力推进交通、电梯、消防、危化品、非煤矿山等重点行业领域安全生产专项整治，严格落实企业主体、属地管理和部门监管责任，坚决防止各类事故发生。争创国家食品安全城市。力争建成无传销城市。加强信访基层基础建设，化解信访突出问题。加强重大事件应急处置。深化“平安芜湖”建设，健全全方位、立体化、信息化社会治安防控体系，推进四级综治（中心）站建设，推进公共安全视频监控建设联网应用，完善矛盾纠纷多元化解机制，积极预防、依法严惩违法犯罪活动。

四、加强政府自身建设

围绕实现皖江崛起排头兵、五大发展先行者目标，全力建设人民满意的政府。

（一）坚持依法行政。始终用法治的理念、思维和方式推动政府工作，严守法律法规规章，提升政府工作人员运用法规解决问题的能力，把政府工作全面纳入法治轨道，做到法无授权不可为、法定职责必须为。认真执行市人大及其常委会的决议和决定，重大决策出台前向市委请示、向人大报告、与政协协商。自觉接受人大监督，主动接受政协监督，认真接受司法监督，广泛接受社会和舆论监督。严格按照市政府工作规则行使政府权力。各议事协调工作机构严格按照各自的议事规则议事决策。落实全市重大事项合法性审查机制和重大执法决定法制审核制度。全面依法推进政务公开。

（二）坚持服务至上。健全“亲切服务”长效机制，做好“双联系”、政风行风评议、“群众满意度”调查工作。深入推进机关效能建设，下大力气解决政务服务意识不强、效率不高、“庸懒散”等问题，对典型事例严肃处理、公开曝光。创新管理方式，建立健全科学、精准考核体系，推进政府服务标准化、高效率。建设“易政网”。完善公共服务清单体系，探索实行市级公共服务事项申报材料“承诺制”和数据共享。扩大政府购买公共服务范围，探索在更多的事务性公共管理服务中引入竞争机制。加快中小企业公共服务体系建设，提升各级企业服务中心的服务水平，整合社会各类服务资源，为广大创业者和中小企业提供更多服务。

（三）坚持廉洁自律。始终把纪律和规矩挺在前面，严格落实“一岗双责”制度，强化廉政风险防范体系建设，织牢织密制度笼子，强化监督，最大限度压缩权力寻租空间。政府机关工作人员带头讲修养、讲道德、讲诚信、讲廉耻，在依法用权、正确用权、干净用权中保持廉洁，在守纪律、讲规矩、重名节中做到自律。深入贯彻中央八项规定、国务院约法三章、省“三十条”精神和市“三十一条”，加大“四风”整治和曝光力度。坚持以零容忍态度惩治腐败，严肃查处公职人员违法违纪行为。统筹推进群众身边腐败问题专项治理三年行动计划，加强村级“小微权力”监督制约。

各位代表！形势催人奋进，奋斗成就梦想。让我们更加紧密团结在以习近平同志为核心的党中央周围，在中共芜湖市委的坚强领导下，切实增强担当精神、责任意识，科学务实，团结拼搏，为建设创新协调绿色开放共享新芜湖而不懈奋斗！
